# Psychological Recovery of “Second Victims” After Adverse Events: Experiences From Generation Z Emergency Nurses

**DOI:** 10.1155/jonm/2257987

**Published:** 2026-03-20

**Authors:** Yue Liu, Yu Zhang, Xiaoxia Zhu, Jingyi Zou, Jiani Zheng, Shuyang Liu, Chunwei Chi, Li Zeng, Jinxia Jiang

**Affiliations:** ^1^ Nursing Department, Shanghai Tenth People’s Hospital, School of Medicine, Tongji University, Shanghai, 200072, China, tongji.edu.cn; ^2^ Department of Nursing, Shanghai Jiguang Polytechnic College, Shanghai, 201901, China; ^3^ Nursing Department, Community Health Service Center of Pengpu New Village Street of Shanghai Jing’an District, Shanghai, 200435, China; ^4^ Central Sterilized Supply Department, First Affiliated Hospital of Naval Medical University, Shanghai, 200438, China; ^5^ Nursing Department, Tongji Hospital, School of Medicine, Tongji University, Shanghai, 200065, China, tongji.edu.cn

**Keywords:** Generation Z, nursing staff, patient safety, psychological recovery, second victim, support programs

## Abstract

**Background:**

The second victim (SV) phenomenon is common among nurses in emergency departments. SVs may experience positive changes and achieve psychological recovery due to new insights or gains, even while enduring second victim syndrome (SVS). Generation Z emergency nurses (ENs) in China possess distinct life experiences compared with previous generations, and their work characteristics influence their psychological recovery experiences.

**Objective:**

This study aimed to investigate the psychological recovery experiences of Generation Z ENs who served as SVs after adverse events.

**Methods:**

This study employed a descriptive phenomenological approach. From April to May 2024, semistructured in‐depth interviews were conducted with eleven Generation Z ENs from a tertiary hospital in Shanghai. The Colaizzi seven‐step framework was employed for data analysis.

**Results:**

Three themes and eight subthemes emerged: shock and stress, reconstruction and remodeling, and gain and growth.

**Conclusion:**

As SVs, positive introspection, resilience mobilization, and the receipt of emotional first aid facilitated the reconstruction and growth of Generation Z ENs. To create effective strategies, managers should prioritize the work characteristics and needs of Generation Z ENs at various stages of psychological recovery, emphasizing self‐efficacy, collective agency, and social capital. The findings of this study are relevant to the development and implementation of future SV support programs.

## 1. Introduction

Adverse events (AEs) are regarded as medical errors that compromise patient safety (PS) and can result in poor health outcomes, fatalities, financial costs, prolonged hospitalization, and a negative image of healthcare professionals [1]. Patients and their families are considered the first victims of AEs. The Institute of Medicine (IOM) issued two landmark reports in 1999 and 2001, titled “To Err Is Human: Building a Safer Healthcare System” and “Crossing the Quality Chasm: A New Health System for the 21st Century,” to underscore the high priority given to AEs and PS. Subsequently, in 2003, the IOM reported “Keeping Patients Safe: Transforming the Work Environment of Nurses,” which emphasized the importance of cultivating positive practice environments to enhance PS [2]. Additionally, the Magnet Recognition Program model, presented by the American Nurses Credentialing Center (ANCC), focuses on empowering nurses in clinical decision‐making, improving the practice environment, promoting mental well‐being, and ensuring PS while fostering professional development [3]. As previous studies have indicated, AEs not only impact PS and recovery but also affect nurses, rendering them second victims (SVs) [4]. However, the public often prioritizes the patient’s perspective when reporting an AE, neglecting the emotional crises faced by nurses. The emergency department (ED) is a critical component of the emergency medical services system (EMSS) and serves as the frontline of hospital emergency care. It is characterized by critical patient conditions, complex diseases, crowding, incomplete patient information, and insufficient human resources [5]. Emergency medical personnel are frequently involved in AEs, such as adverse treatments, accidents, and occupational exposures, as well as witnessing AEs and experiencing near‐error events. Emergency nurses (ENs), who have the most patient contact within EMSS, are particularly vulnerable to SVs due to their high‐intensity, high‐risk, high‐workload, and high‐expectation work environment. SVs often experience physical or psychological symptoms following such events, including self‐blame, embarrassment, anxiety, depression, muscle tension, increased respiratory rate, difficulty concentrating, sleep disturbances, and gastrointestinal distress, collectively referred to as second victim syndrome (SVS). Some ENs experiencing SVS ultimately face negative psychological outcomes (post‐traumatic stress disorder, burnout), negative physical outcomes (chronic illness), and negative career outcomes (quitting, defensive healthcare behaviors) [1]. This situation contributes to a shortage of nursing human resources, which is ultimately detrimental to PS and recovery.

**FIGURE 1:**
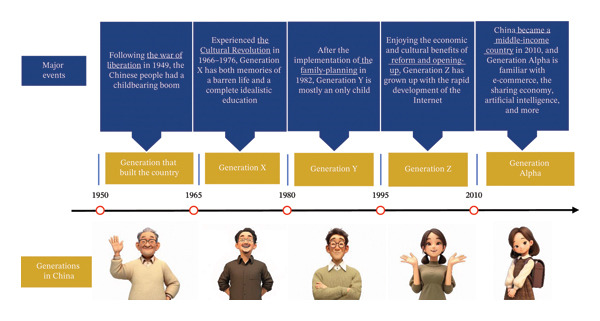
Generational division in China.

**FIGURE 2:**
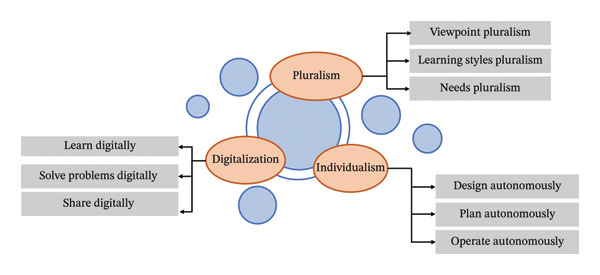
Work characteristics of Generation Z in China.

With workforce turnover, Generation Z nurses (born between 1995 and 2009) are entering and increasingly dominating the nursing profession. According to the China Health Statistics Yearbook issued by the National Health Commission of the People’s Republic of China (2022), 67% of the nurses are under the age of 34 [6]. Born in the “volatile, uncertain, complex, ambiguous” (VUCA) era, most of China’s Generation Z grew up in 4‐2‐1 families (four grandparents, two parents, and one child) (Figure 1). Characterized by “individualism, digitalization, and pluralism” (Figure 2), the second generation of China—where both parents are the first generation of only children—enjoys considerable material wealth, receives a better education, and utilizes the Internet extensively [5]. In China, ED is one of the areas with the highest number of Generation Z nurses. On one hand, the ED is a compulsory department where newly registered Generation Z nurses must complete two years of standardized training. This training includes rotations in internal medicine, surgery, ED, obstetrics and gynecology, pediatrics, operating rooms, and other relevant areas. On the other hand, the unique characteristics of the ED, such as high risk and high pressure, contribute to a high turnover rate among ENs, with new recruits typically being younger. Notably, despite the two years of standardized training with an emphasis on safety education, the high number of acutely and critically ill patients, high mortality rates, intense work demands, and high risk of occupational exposure in the ED make Generation Z ENs—who often lack clinical experience and undergo transformational shock—more susceptible to AEs and thus more likely to become SVs [1].

As previously noted, SVs experience negative emotions. However, several studies have indicated that some healthcare professionals who become SVs report experiences of psychological recovery and positive changes, which refers to the trauma‐change‐recovery process [7]. Scott proposed that SVs’ psychological recovery after AEs involves six stages: chaos and accident response, intrusive reflections, restoring personal integrity, enduring the inquisition, obtaining emotional first aid, and moving on [4]. For instance, a Danish study found that 50%–65% of the professionals indicated that after encountering AEs, they reflected more on the meaning of life and became better healthcare professionals [8]. Most research on SVs has been conducted in Western countries, highlighting a significant research gap due to the lack of studies based in Asian contexts. The unique emergency environment and work characteristics of Generation Z in China may significantly influence the experiences of SVs. Therefore, the purpose of this study was to investigate the psychological recovery experiences of Generation Z ENs as SVs after AEs within the healthcare environment in China.

## 2. Methodology

### 2.1. Research Design

A descriptive phenomenological approach was employed to analyze the experiences and perceptions of psychological recovery for Generation Z ENs as SVs after AEs. This approach, along with semistructured in‐depth interviews, is appropriate for exploring complex phenomena such as human emotions and experiences [9].

### 2.2. Participants

Purposive sampling was utilized to recruit Generation Z ENs from a tertiary hospital in Shanghai. The inclusion criteria were as follows: nurses who (1) worked in the ED and have been engaged in emergency nursing > 1 year; (2) have experienced AEs within the last 6 months; (3) were born between 1995 and 2006; and (4) demonstrated a willingness to participate in this study. The exclusion criteria included major adverse life events occurring within the past 3 months. The study included 11 Generation Z ENs, aged 25–29 years (mean 27.36 ± 1.36), with 3.64 ± 2.25 years of ED work experience (Table 1).

**TABLE 1:** Characteristics of participants (*n* = 11).

No.	Sex	Working years in the ED	Age	Education level	Marital status	Types of AEs	Disposal results
N1	Female	2	27	Bachelor’s degree	Single	Unplanned extubation	Point deduction from the Nursing Quality Control Group and financial penalties
N2	Female	9	29	Diploma	Married	Patient falls	Verbal criticism
N3	Female	4	28	Bachelor’s degree	Single	Pressure injuries	Point deduction from the Nursing Quality Control Group
N4	Male	2	27	Bachelor’s degree	Single	Unplanned extubation	Point deduction from the Nursing Quality Control Group and financial penalties
N5	Female	3	28	Bachelor’s degree	Single	Medication error	Financial penalties
N6	Female	2	25	Diploma	Single	Unplanned extubation	Point deduction from the Nursing Quality Control Group and verbal criticism
N7	Female	6	29	Bachelor’s degree	Single	Patient falls	Point deduction from the Nursing Quality Control Group
N8	Female	2	26	Bachelor’s degree	Single	Missing accident of patient	Verbal criticism and financial penalties
N9	Female	2	26	Bachelor’s degree	Single	Drug extravasation	Financial penalties
N10	Female	5	29	Bachelor’s degree	Married	Medication error	Financial penalties
N11	Female	3	27	Bachelor’s degree	Single	Patient falls	Verbal criticism

*Note:* The Nursing Quality Control Group consisted of the chief nurse, nurse managers, and clinical nursing specialists. The nursing quality control program’s formulation and execution, as well as the assessment and enhancement of nursing care, are under its responsibility.

### 2.3. Data Collection

The study followed a semistructured interview framework and utilized in‐depth interviews to collect qualitative data. The semistructured interview questions (Table 2) were derived from previous studies and facilitated a comprehensive presentation of the participants’ views [4, 8, 10]. Although semistructured interviews allow for some flexibility, a predesigned interview framework may carry potential guiding biases [11]. Therefore, some open‐ended questions (such as No. 6) were utilized to encourage Generation Z ENs to express their experiences freely, uncover in‐depth information, and avoid preconceived biases. To assess the validity of the interview questions, the researcher conducted preinterviews with two Generation Z ENs. These preinterviews were solely used to test the validity of the questions and were not included in the data analysis. Between April and May 2024, the interviews were conducted in a quiet conference room, with each interview lasting 40–50 min. The duration of the interviews varied depending on the information presented, the participants’ responses, and their willingness to continue. Finally, 11 Generation Z ENs were interviewed for 522 min (mean = 47.45 min). Two experienced researchers (J.X.J. and Y.L.) conducted the interviews. The interviews were recorded using a digital voice recorder, which ensured precise capture of the content. Although there are no established principles regarding sample size in qualitative research, data collection was considered complete when no further data or new concepts related to the topic were obtained [11]. Following this guideline, the sample size and domain size were estimated at the saturation point (11 participants). Each participant received a bottle of shower gel as a small gift, agreed to be contacted again, and provided their email address and cell phone numbers.

**TABLE 2:** The semistructured interview questions.

No.	Question
1	How did you feel after AE?
2	How did AE influence you?
3	How did you cope with these disturbances?
4	What kind of support did you get during AE?
5	What kind of positive changes did you make following this AE?
6	What other experiences and feelings do you have?

### 2.4. Ethical Considerations

The Institutional Review Committee of Shanghai Tenth People’s Hospital approved the study (no. 23KN25). Generation Z ENs signed the written informed consent form that followed the principles of the Helsinki Declaration before being interviewed. Participants were informed of the purpose of the interview prior to its commencement and were allowed to withdraw from the study at any time. If names were mentioned during the interviews, they were replaced with numeric codes to ensure privacy, such as “N1.” Participants were advised that if they experienced emotional distress while reviewing AEs, a hospital psychologist was available for consultation and research team could provide emergency psychological intervention. Participants were given time to relax and decide whether to continue with the interview. To ensure participants’ psychological safety and confidentiality, all interviews were conducted in a quiet conference room. None of the participants required additional assistance [12].

### 2.5. Data Analysis

**FIGURE 3:**
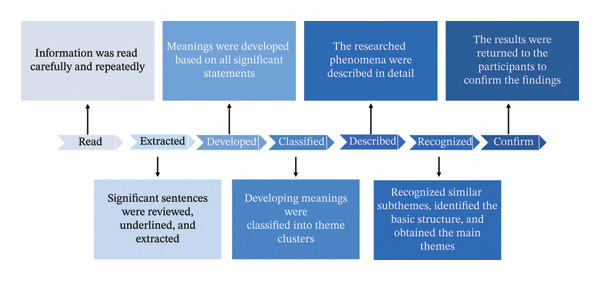
The Colaizzi seven‐step method.

There was no predetermined theoretical framework; all codes were generated immediately from the data. Microsoft Word and Excel Version 16.0 were used for data management. Following the recommendations of DeCuir‐Gunby et al. [13], the researcher (J.X.J.) developed a preliminary codebook and clarified definitions by reading the complete transcripts and coding three transcripts. The codebook was sufficiently flexible and was continuously updated. In this study, two researchers (J.X.J. and Y.L.) chose not to use any software tools for qualitative data analysis, opting instead for manual coding. The Colaizzi seven‐step method was employed, which was an inductive thematic analysis (Figure 3) [9]. To avoid interference with the study results, the researchers “bracketed” or “put in brackets their own ideas and preconceptions under consideration” as much as possible. At each phase of the research process, reflexivity was pursued [14]. Data triangulation was conducted during weekly research team meetings. If there were any disagreements, a more experienced and knowledgeable independent researcher adjudicated.

In the first phase, J.X.J. and Y.L. transcribed the data within 24 h of the interviews. Transcripts were peer‐checked by another researcher (L.Z.) to ensure accuracy. The information obtained was read carefully and repeatedly.

In the second phase, significant sentences about Generation Z ENs’ experiences of psychological recovery as SVs after AEs were reviewed, underlined, and extracted.

In the third phase, meanings were developed based on all significant statements and reviewed by two researchers with extensive experience in qualitative research.

In the fourth stage, developing meanings were categorized into theme clusters based on the similarity of semantic content. During this process, researchers compared the theme clusters with the original data to ensure consistency and repeated these processes several times.

In the fifth stage, eight subthemes were identified to describe Generation Z ENs’ experiences of psychological recovery as SVs after AEs, and these were described in detail.

In the sixth stage, similar subthemes were categorized into larger clusters, resulting in three main themes.

In the seventh stage, a one‐page summary was given to the participants, outlining the themes and subthemes, along with a simple explanation of the main findings. Participants strongly resonated with the results, and no further modifications were made.

### 2.6. Study Rigor

**FIGURE 4:**
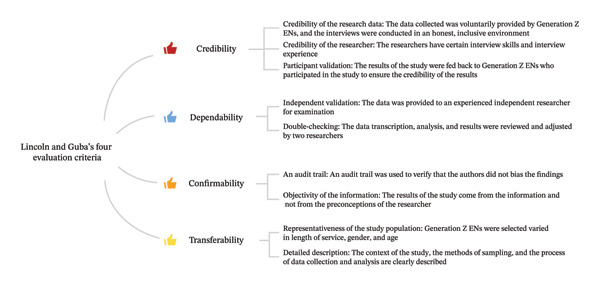
Lincoln and Guba’s four evaluation criteria.

Methods and findings were presented in a well‐organized manner, guided by the Consolidated Criteria for Reporting Qualitative Research guidelines [11]. Lincoln and Guba’s four evaluation criteria were utilized to establish the credibility, dependability, confirmability, and transferability of the research (Figure 4) [15].

### 2.7. Reflexivity

This study was conducted by a team of nine Chinese nursing scholars deeply rooted in collectivism and Confucian cultural contexts. This background affords the team a nuanced understanding of how such a cultural environment shapes the psychological recovery of Generation Z ENs who become SVs following AEs. The corresponding author (J.X.J.), a nursing manager and researcher with 21 years of experience, brings extensive expertise in qualitative methodology and substantial practical experience in managing AEs and medical disputes, providing both methodological rigor in research and a macro‐level perspective on PS. The first author (Y.L.) contributes a vital “insider” perspective, having personally experienced an AE and its accompanying direct psychological sensations, which deepens empathy for the feelings of SVs [16]. Furthermore, the inclusion of three Generation Z researchers on the team helped bridge generational gaps, facilitating a more accurate interpretation of peer‐specific workplace metaphors. Meanwhile, to mitigate the risk of subjective projection in such perspectives, the team employed several bracket strategies, including independent dual coding, collaborative team analysis, and reflexive journaling. The research was guided by the core principles of “Just Culture” and humanistic care, firmly believing that the well‐being of healthcare providers is fundamental to PS [17]. Throughout the process, the team adhered to strict ethical protocols and objective data coding practices to ensure an authentic representation of the unique psychological recovery experiences of Generation Z ENs as SVs.

## 3. Result

**FIGURE 5:**
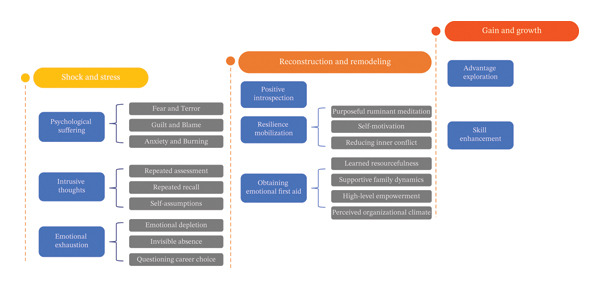
Themes and subthemes.

Three themes and eight subthemes were extracted (Figure 5): (a) shock and stress, (b) reconstruction and remodeling, and (c) gain and growth. The themes, along with the number of participants who mentioned each subtheme and the frequency of their mentions, are presented in Table 3.

**TABLE 3:** Frequency of themes and subthemes identified.

Themes	Subthemes	Number of participants who mentioned subthemes (*n*)	Percent of participants who mentioned subthemes (%)
Shock and stress	Psychological suffering	11	100.00
	Intrusive thoughts	6	54.55
	Emotional exhaustion	7	63.64

Reconstruction and remodeling	Positive introspection	8	72.73
	Resilience mobilization	10	90.91
	Obtaining emotional first aid	9	81.82

Gain and growth	Advantage exploration	5	45.45
	Skill enhancement	8	72.73

### 3.1. Shock and Stress

Shock refers to the negative responses that Generation Z ENs suffer immediately or shortly after AEs, which include both psychological and physical outcomes. At this stage, Generation Z ENs exhibited psychological suffering, intrusive thoughts, and emotional exhaustion, all of which are typical manifestations of SVS [4]. Stress is a heavy burden that follows and continues.

#### 3.1.1. Psychological Suffering

Generation Z ENs reported varying degrees of psychological suffering due to poor patient health outcomes, a punitive safety culture, complex ED environments, and strained nurse–patient interactions, which are the direct experience of the shock and stress. According to research, Generation Z nurses are significantly more stressed than their Generation X and Generation Y counterparts due to factors such as heavy workloads, occupational instability, and low compensation [18]. When serving as SVs, increased stress exacerbates their distress, making psychological suffering especially severe among Generation Z ENs (*n* = 11). One participant described her fear and terror:

“When I realized the patient was missing, I shook with fear.” (Participant 8).

Another participant expressed both guilt regarding the impact of the AE on the patient and self‐blame for the consequences of the demerit system in the ED:

“I felt so guilty and self‐blamed that I was afraid to face the patient and family and could only pray for his safety. I’m also afraid of quality and safety demerits, and even more worried that the nurse manager will be implicated. I’m clipping the wings of the team, and it makes me very uneasy.” (Participant 1).

A different participant stated that any reporting, reviewing, or related processes concerning AEs made him feel anxious and distressed:

“I couldn’t hold my head up when the higher level organized the Root Cause Analysis (RCA). To protect me, the nurse manager helped me debrief the event, but I felt like I was on pins and needles with a million eyes watching me. That emotion may continue forever.” (Participant 4).

#### 3.1.2. Intrusive Thoughts

Some participants (*n* = 6) reported intrusive thoughts following AEs, which exacerbated the shock at the cognitive level, typically characterized by uncontrollability, repeatability, and negativity. Although Generation Z ENs are more open about mental health (with lower stigma) and more attentive to it than previous generations [18], nurses from all generations who experience AEs may have similar psychological responses—such as repeated assessment and recall—that form the basis of intrusive thoughts. One participant expressed fear of repeating the same mistake, especially when faced with a clinical situation similar to the AE:

“As the saying goes, ‘Once bitten, twice shy.’ I was scared of intubated patients for a long time. I would repeatedly visit him out of fear that the patient might pull out the tube.” (Participant 6).

Another participant recounted AEs repeatedly and expressed concern about the potential deterioration of the patient’s condition as well as career‐related repercussions:

“When I have to care for a patient with the same condition, I recall that day and imagine all the possible terrible consequences. It’s an indescribable feeling.” (Participant 7).

Post‐AE, participants felt discriminated against by colleagues and sometimes developed self‐assumptions that extended beyond work:

“It always feels like people are secretly saying bad things about me, and it feels like I’m being labeled.” (Participant 2).

#### 3.1.3. Emotional Exhaustion

Emotional exhaustion is the result of psychological suffering and intrusive thoughts caused by shock and stress. Although nurses of all generations may experience this process, Generation Z (*n* = 7) may experience increased emotional exhaustion compared with the compliant and easily satisfied Generation X, possibly due to reality shock from their unique work characteristics and traditional nursing culture [19, 20]. Generation Z ENs experienced fatigue, tension, and anxiety after AEs, leading to emotional depletion. Emotional depletion is a significant factor and a core component of burnout. Two participants developed an intention to leave due to burnout and a lack of professional efficacy:

“I feel very frustrated and have lost my confidence.” (Participant 5).

“I feel particularly worn out and on the verge of depression. Due to the patient’s uncooperative family, this event will happen. I’m especially upset and don’t want to be a nurse.” (Participant 3).

Invisible absence occurs when an employee continues to work despite being ill but is inefficient and unproductive. One participant experienced invisible absence due to emotional depletion:

“I’m particularly anxious. Sometimes I needed a colleague to remind me to proceed during team first aid because I was distracted and overwhelmed.” (Participant 7).

Another participant even questioned her career choice:

“I felt like I had a stain on my career, and I didn’t even feel like I was suitable for this job anymore.” (Participant 2).

### 3.2. Reconstruction and Remodeling

Reconstruction and remolding refer to the processes by which Generation Z ENs begin to rebuild a sense of control and basic order after suffering the initial shock and stress of AEs, eventually achieve deeper psychological recovery. When Generation Z ENs understood that they could not change the realities, they began to reconstruct and remold their cognition, coping abilities, and coping resources through positive introspection, mobilizing resilience, and obtaining emotional first aid.

#### 3.2.1. Positive Introspection

Positive introspection allows individuals to examine their behaviors, attitudes, and values to promote positive change and progress, which is the reconstruction and remolding of cognition. It was found that the introspection of Generation Z ENs (*n* = 8) extends beyond examining personal capabilities or responsibilities; they combine personal experiences with broader organizational and environmental systems. One participant shared her AE experiences with team members to help others avoid similar mistakes:

“Our team reviewed workflow issues. I also realized that ‘three inspections and seven verifications’ is not just a slogan but an important patient safety guideline.” (Participant 10).

Another participant reflected on his personal and professional life from pluralistic viewpoints:

“As the old saying goes, ‘A fall into the pit, a gain in your wit,’ and I now have a deeper appreciation of this saying.” (Participant 8).

A different participant gained practical wisdom from the AE experience:

“I paid more attention to the weak points in my work. I consider it from multiple dimensions: patients, machines, raw materials, methods, and environment. I put into practice the training I usually receive to ensure patient safety.” (Participant 1).

#### 3.2.2. Resilience Mobilization

Resilience refers to the ability to continuously improve work performance, adapt to adversity, and manage negative emotions [10]. The mobilization of resilience is the reconstruction and remolding of coping abilities. According to one study, Generation Z has active minds and is adept at leveraging resources, whether online or offline, frequently discovering approaches that work best for them. As a result, compared with previous generations, they (*n* = 10) are better able to motivate themselves and develop resilience [5]. Most participants fully mobilized their resilience and utilized purposeful rumination meditation for mental adjustment, alleviating emotional distress, facilitating a shift in positive thinking, and ultimately resolving the crisis. Due to the digital nature and pluralism of their environment, one participant engaged in purposeful rumination meditation using various applications and internet platforms:

“I enjoy listening to audiobooks on Himalaya. I really like the Antifragile: Things That Gain from Disorder, which teaches me how to turn adversity into opportunities for progress.” (Participant 6).

After experiencing the Craze for the Postgraduate Entrance Examination, Civil Servant Fever, the COVID‐19 epidemic, and employment pressure, Generation Z ENs have become more self‐motivated. The self‐motivation approaches of Generation Z differ significantly from those of earlier generations, combining digital technologies, social networking, and psychological concepts. Two participants reported that self‐motivation helps them maintain a positive attitude and strong beliefs when facing challenges:

“It was hard, but I kept going. I wrote my story in ‘The Little Red Book’ to motivate myself and received support from online friends.” (Participant 3).

“Words are powerful. Avoid discussing annoying and frustrating feelings. Allow yourself to go to seed for a few days when in pain and believe, ‘It is not a big problem; I can handle it.’ Don’t let your mind destroy you; being pessimistic wastes your life.” (Participant 9).

The individualism of Generation Z ENs is neither collectivism nor traditional selfishness but rather the work characteristic of autonomous. Individualism and immediate action enable them to quickly initiate coping strategies, reduce interference from external evaluations, and transform individualism into an engine of resilience:

“I was depressed for a while, so I returned to my hometown and took a trip to treat myself.” (Participant 1).

#### 3.2.3. Obtaining Emotional First Aid

Most participants received significant emotional first aid from four aspects: learned resourcefulness, supportive family dynamics, high‐level empowerment, and organizational climate, which is the reconstruction and remolding of coping resources. In a study of how nurses from different generations cope with stress and their coping strategies, Generation X tended to engage in avoidance behaviors [18], while Generation Z ENs (*n* = 9) in the interviews demonstrated more proactive behaviors in seeking coping resources. Resourcefulness refers to the ability to solve daily difficulties independently and seek external support when needed. Resourcefulness can alleviate the suffering of Generation Z ENs as SVs and maintain their psychological and physical well‐being [21]. One participant described:

“Self‐reliance beats reliance on others. I use a Gantt chart to visualize future plans to reduce anxiety and remind myself there’s more to do.” (Participant 2).

Supportive family dynamics view the family as an interactive system that reflects members’ behaviors, psychology, communication, and the family’s relationship with the outside world [22]. The family serves as an individual’s primary environment and is a core concept in traditional Chinese Confucian thought. As previously stated, Generations X and Y grew up in a time of material relative deprivation, with the traditional intergenerational covenant tied to survival needs (e.g., “raising children to provide for old age”). Generation Z ENs, on the other hand, often perceive supportive family dynamics as spiritual pillars in their reconstruction and remolding process [7]. One participant described how supportive family dynamics helped her detach from SVS and gain emotional value:

“My family often encouraged me and gave me specific advice.” (Participant 5).

High‐level empowerment and transformational leadership meet the individualism and pluralism of Generation Z ENs, not only improving their emotional support and professional performance but also enhancing their growth potential. One participant stated that empowerment enhanced self‐efficacy and met diverse needs:

“The nurse manager asked me to suggest recommendations for workflow optimization. I received her praise when I drew the suggestions into a mind map. I felt the sincerity and warmth of the manager and my own value.” (Participant 7).

Organizational climate refers to the atmosphere of the work environment, encompassing individual, team, and management climates [23]. Most Generation Z individuals in China are only children, so academic pressure and a lack of companionship limit their external social contacts, resulting in strong social and emotional needs [5]. One participant noted that a positive organizational climate could reduce the shock of AEs and meet their emotional needs:

“I felt it would be challenging for me to step out of the shadows, but the nurse manager and my colleagues helped me a lot, and I appreciate it.” (Participant 8).

### 3.3. Gain and Growth

Gain and growth refer to the positive changes in Generation Z ENs’ spiritual and behavioral dimensions after experiencing AEs. Specifically, this process includes the discovery of strengths and the improvement of skills.

#### 3.3.1. Advantage Exploration

Generation Z, also known as “digital natives,” are both technology consumers and creators. Compared with previous generations, Generation Z ENs expect frequent, timely, and supportive feedback to explore their advantage and gain confidence (*n* = 5) [24]. One participant utilized digital advantages such as short films, animations, and artificial intelligence (AI) chatbots in educational activities related to AEs:

“I produced Shanghai and Mandarin versions of the easy‐to‐understand ‘fall prevention’ film, which patients and their families complimented.” (Participant 4).

Generation Z ENs are beginning to emerge as a dominant force in nursing research. One participant used research to enhance nursing management:

“After reflecting, I wrote meta‐analyses on catheter management from an evidence‐based perspective. Research could help improve patient safety. I feel very accomplished and confident.” (Participant 5).

#### 3.3.2. Skill Enhancement

As digital natives, Generation Z enhances their skills through learning that emphasizes speed, nonlinear processing, digitalization, pluralism, individualism, and personalized learning, which is consistent with previous research [20]. Unlike Generation Y, who prefers to read physical books, Generation Z ENs (*n* = 8) do not rely solely on traditional learning materials. Instead, they actively learn professional skills through various applications (e.g., Bilibili and YouTube) and achieve digital knowledge transformation [25]. Two participants improved their nursing practice skills by employing diverse styles:

“I studied many videos on skin management on ‘Bilibili,’ and they were quite helpful.” (Participant 6).

“I understand emergency care risks better, and I have improved my ability to anticipate and assess areas of weakness. (Participant 3).”

One participant converted disease knowledge into popular science content, improving health promotion skills:

“I successfully delivered a scientific lecture on the mechanism of action of vasoactive drugs and the prevention of complications using illustrated PowerPoint slides. Patients praised my efforts.” (Participant 11).

## 4. Discussion

This qualitative research investigated the psychological recovery experiences of Generation Z ENs in China as SVs after AEs. Three themes were extracted: shock and stress, reconstruction and remodeling, and gain and growth. This somewhat parallels Scott’s psychological recovery framework for SVs [4]. However, the details of psychological recovery for Generation Z ENs differ due to cultural differences and management practices.

In this study, Generation Z ENs in China as SVs experienced psychological suffering, intrusive thoughts, and emotional exhaustion as the norm rather than the exception. This aligns with Scott’s framework regarding the early stages of psychological recovery (chaos and accident response, intrusive reflections) [4]. Generation Z ENs reported feeling overwhelmed by negative emotions such as fear, guilt, and anxiety. Individuals experiencing an AE endure distressing experiences characterized by intrusive thoughts, which motivate them to respond in various ways. Generation Z ENs often find themselves in a state of heightened physiological arousal, employing coping strategies to mitigate intrusive thoughts, including avoidance behaviors (e.g., emotional depletion, invisible absence), which are associated with Asian culture (e.g., Daoist Reclusive Philosophy) [26]. Furthermore, the study found that the shock and stress experienced by Generation Z ENs as SVs are linked to their generational characteristics. First, self‐motivated growth and an expectation gap (i.e., the discrepancies between the professional aspirations of highly educated Generation Z ENs and their low self‐efficacy) may increase Generation Z ENs’ psychological suffering. Second, Generation Z, who are digital natives, are exposed to a large amount of information and demand instant feedback; however, this also causes unavoidable information overload and a digital work environment (e.g., WeChat notifications and online work group communications anytime, anywhere), which may act as recurring triggers. Third, pluralism (particularly cultural, identity, and value pluralism) makes Generation Z ENs extremely vulnerable to traditional organizational punishment and a “shame and blame” culture, which can lead to additional moral quandaries and psychological suffering, exacerbating emotional exhaustion. Although shock and stress (psychological suffering, intrusive thoughts, and emotional exhaustion) provided plenty of negative experiences for Generation Z ENs, they were also the primary motivators for Generation Z’s following actions and psychological recovery. Generation Z ENs were struggling to cope with the consequences of AEs, process relevant information, and begin to seek resources. At this stage, reducing harm to patients from AEs is the primary concern. In addition, managers should create an understanding healthcare environment and supportive culture while avoiding stigma and horizontal violence, such as bullying or shaming. It is important to note that the potential for growth of Generation Z ENs should be emphasized; premature intervention is discouraged, as it may waste valuable resources and interfere with SVs’ natural recovery process [27].

According to our findings, while AEs can be shocking and stressful, Generation Z ENs still experienced positive changes and accomplished the reconstruction and remolding of cognition, coping abilities, and resources. Conservation of Resource Theory (COR) suggests that when individuals lose resources, they will take steps to conserve existing resources while also seeking to acquire new ones. More altruistic behavior—achieved through the reciprocal mechanism of social exchange—often occurs when employees experience severe negative emotions [28]. Research indicates that cognitive appraisal is the most important factor in the stress coping process [29]. Positive introspection, the initial step in reconstruction and remodeling, facilitates cognitive appraisal (e.g., analyzing the situation, discovering meaning, and reassessing). For instance, Generation Z ENs redefine the meaning of AEs to learn from their mistakes and prevent similar incidents from occurring in the future.

Another important finding is that resilience is mobilized under shock and stress and is enhanced by purposeful rumination meditation, self‐motivation, and the reduction of inner conflict, which were not mentioned in Scott’s psychological recovery framework for SVs [4]. According to the International Council of Nurses (ICN), enhancing resilience not only benefits nurses’ physical and mental health but also contributes to PS and organizational stability [5]. First, Generation Z ENs effectively employed digital technologies to create instant feedback for psychological adjustment, replacing conventional “rumination meditation.” This is crucial for maintaining psychological well‐being and mobilizing resilience. Next, viewpoint pluralism and digital attributes such as value reorientation, rational use of online platforms, and stress regulation help Generation Z ENs achieve self‐motivation and resilience mobilization. Again, individualism allows Generation Z ENs to disregard others’ opinions, reduce internal conflict, and quickly implement coping strategies. This is a manifestation of self‐confidence, which is associated with wealthy living conditions and high‐quality education, and it contributes positively to the resilience of Generation Z ENs [5].

The triadic reciprocal causation of social cognitive (SC) theory identifies self‐efficacy and collective agency as critical to overcoming adversity and building resilience. Self‐efficacy is the degree of an individual’s belief in their ability to accomplish a behavior in a given domain. Collective agency, as an extension of individual mobility, represents a collective force in which individuals share common beliefs to achieve desired outcomes [30]. First, considering the work characteristics of Generation Z ENs, managers can enhance these traits by adopting transformational leadership, improving personal‐work fit, reducing work intensity, and particularly by fostering empowerment, which are effective strategies for enhancing self‐efficacy and mobilizing resilience [31]. Second, plans to enhance collective agency should be encouraged, focusing on the team level. This includes improving team resilience, fostering constructive organizational communication, establishing knowledge‐sharing mechanisms, and building conflict resolution skills through interactive workshops (e.g., 1.5 h sessions). Among these, team resilience serves as a protective factor for individual resilience. Team members extend their personal resilience to the group level through interpersonal interactions when facing adversity [5].On the other hand, given the importance of collective agency in nursing teams, the social capital of the practice team is critical. COR emphasizes that a positive healthcare environment generates significant social capital [28]. For instance, the National Academy of Medicine has integrated social capital into the Action Collaborative for Clinician Well‐Being and Resilience to improve practice environments for healthcare providers [30]. The Guiding Opinions on Strengthening the Nursing Workforce and Optimizing Nursing Services emphasized the importance of social capital and improving safeguards for salaries, staffing, and title promotion [32]. Furthermore, the Chinese government established nursing as a state‐controlled distribution profession in 2024 to foster its development and guarantee PS [33]. A positive healthcare environment, psychological health assistance, individualized welfare policies, and professional development support provide Generation Z ENs with a higher level of social capital and better mentoring. These measures will help them develop a sense of self‐efficacy and increased resilience, ultimately making them the backbone of their teams.

**FIGURE 6:**
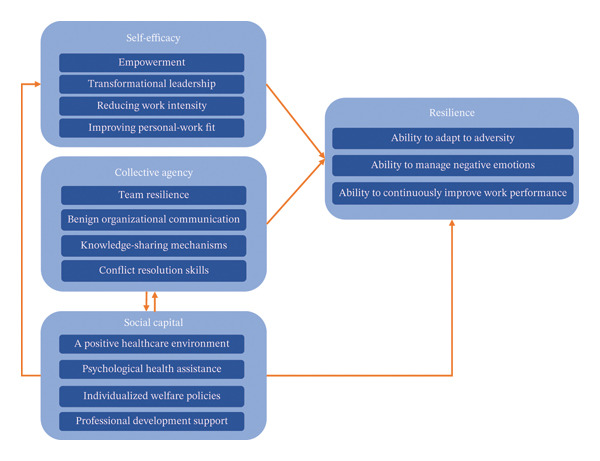
Intervention proposals for Generation Z ENs’ resilience. Notes: This diagram illustrates self‐efficacy, collective agency, and social capital related to the resilience of Generation Z ENs, along with the relationships among them. Unidirectional arrows between labels represent the sequence of events and/or the direction of influence. Bidirectional arrows indicate reciprocal influence relationships between them.

In the process of psychological recovery, SVs obtained emotional first aid, which was particularly prominent among Generation Z ENs in this study. Sources of emotional first aid include learned resourcefulness, supportive family dynamics, high‐level empowerment, and organizational climate. This contrasts with Scott’s framework [4], which describes two separate stages (restoring personal integrity and obtaining emotional first aid). However, in this study, these stages overlap and occur simultaneously. The individualism of Generation Z is not “isolation” in the traditional sense, but rather an enhancement of resourcefulness powered by technology. Talking about AEs with family members is an important coping strategy for Generation Z ENs. Supportive family dynamics alleviate suffering and reduce emotional stress by providing support and opportunities for learning. Furthermore, high‐level empowerment, transformational leadership, and organizational climate perceptions not only provide Generation Z ENs with emotional first aid but also increase their self‐efficacy and resilience. As described in SC theory, resources derived from social relationships (e.g., family, leadership, and organization) are critical in developing resilience and facilitating psychological recovery among Generation Z ENs [5]. Figure 6 provides an overview of how self‐efficacy, collective agency, and social capital are related to the resilience of Generation Z ENs, as well as their interrelationships.

**FIGURE 7:**
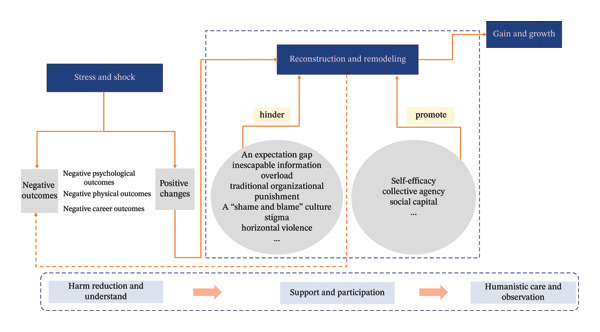
Intervention proposals for Generation Z ENs’ psychological recovery. Notes: This diagram illustrates three themes of Generation Z ENs’ psychological recovery experiences, along with their dynamic interconnections and intervention proposals. Unidirectional solid arrows between labels represent the sequence of events and/or the direction of influence. Unidirectional dashed arrows indicate secondary or negative pathways.

Scott described three possible outcomes for SVs: dropping out, surviving, or thriving [4]. This study found that Generation Z ENs experienced gain and growth, as reflected in their advantage exploration and skill enhancement. First, Generation Z ENs are typically versatile and think innovatively, so it is important to take advantage of their strengths and provide instant feedback. For example, intergenerational reverse mentoring not only brings new viewpoints, new technologies, and new learning perspectives to Generations X and Y but also enhances Generation Z’s self‐efficacy and sense of belonging. Second, since Generation Z ENs prefer concrete examples and visual feedback, sufficient targeted learning opportunities and educational platforms should be provided. TikTok microvideo training, virtual reality of AEs, and table‐top exercise are all excellent choices [1]. On the other hand, even though Generation Z ENs have been working to overcome the effects of AEs, emotional trauma still persists, albeit less pronounced than in the early stages [4]. Therefore, humanistic care and necessary observation, such as wearing visual stress detection devices, should be extended to Generation Z ENs, regardless of their stage of recovery. Notably, the prevention of AEs must remain a priority. Advocate that all managers in healthcare systems should collaborate with healthcare providers to develop an integrated safety management system that combines safety culture, mentorship, technology, support strategies tailored to multigenerational nurses, and standardized methods for interdisciplinary teamwork. Figure 7 provides an overview of the intervention proposals.

## 5. Conclusion

AEs are unavoidable. When errors occur, Generation Z ENs are affected and become SVs. Positive introspection, resilience mobilization, and obtaining emotional first aid assist Generation Z ENs in reconstructing and remodeling, leading to gain and growth. This highlights the necessity for managers to tailor interventions to the specific needs of Generation Z ENs at various stages, with an emphasis on self‐efficacy, collective agency, and SC. The ultimate goal is to ensure PS, promote the physical and psychological well‐being of Generation Z ENs, and enhance their retention. Simultaneously, this provides a valuable foundation for future research focused on developing and implementing SV support programs.

## 6. Study Limitations

This study was conducted at a tertiary hospital in Shanghai. First, in recent years, the hospital has primarily recruited Generation Z ENs from undergraduate institutions, rarely recruiting nurses from vocational colleges and not recruiting nurses with secondary diploma education. Second, there was only one male and two married participants, which might be attributed to the existing age and gender mix of China’s emergency nursing industry. These factors may cause a lack of diversity in the current sample. Third, due to ethical constraints along with worries that collecting highly sensitive data would likely result in high refusal rates or data distortion, we were unable to directly gather indicators reflecting nurses’ economic status and risk coping abilities, such as the salary grade, personal assets (e.g., purchased houses or cars locally), and Shanghai household registration. To mitigate this limitation, this study used proxy variables such as “education level,” “age,” and “working years in the ED.” Within the hospital system in China, “education level” is a key indicator of professional status and earning potential. “Age” and “working years” are often correlated with nurses’ job control and risk coping abilities, which may indirectly influence psychological recovery [34]. Finally, although all participants belonged to Generation Z, statistical comparisons between different age subgroups (e.g., early Generation Z and late Generation Z) were not carried out due to sample size constraints. Age and work experience have a significant impact on nurses’ psychological recovery abilities; analyzing this large generational cohort as a single entity may obscure internal differences. Future quantitative research with larger sample sizes could provide more detailed insights into these critical factors. Furthermore, the methodological approach to determining data saturation in this study warrants refinement. Although recruitment was ceased after 11 interviews due to the absence of new themes or codes emerging during the analysis, this determination was based on initial saturation. We recognize that a more methodologically rigorous criterion—continuing sampling until at least three consecutive interviews yield no new insights—provides stronger justification for achieving saturation [35]. While the current study design did not permit retrospective application of this decision, adopting such a criterion in future investigations is recommended. This would reduce the potential influence of chance and strengthen the robustness of the findings. However, this study still provides valuable information for future research on the psychological recovery of Generation Z ENs as SVs after AEs.

## Funding

This work was supported by the Shanghai Science and Technology Commission’s 2024 Soft Science Research Project for Science and Technology Innovation Action Plan (Project no. 24692112300).

## Conflicts of Interest

The authors declare no conflicts of interest.

## Data Availability

Due to ethical reasons, the raw data would remain confidential and would not be shared. The processed data that support the findings of this study are available from the corresponding author upon reasonable request.
